# Approximate Exponential Integrators for Time-Dependent Equation-of-Motion Coupled Cluster Theory

**DOI:** 10.1021/acs.jctc.3c00911

**Published:** 2023-12-12

**Authors:** David B. Williams-Young, Stephen H. Yuwono, A. Eugene DePrince III, Chao Yang

**Affiliations:** † Applied Mathematics and Computational Research Division, Lawrence Berkeley National Laboratory, Berkeley, California 94720, United States; ‡ Department of Chemistry and Biochemistry, Florida State University, Tallahassee, Florida 32306, United States

## Abstract

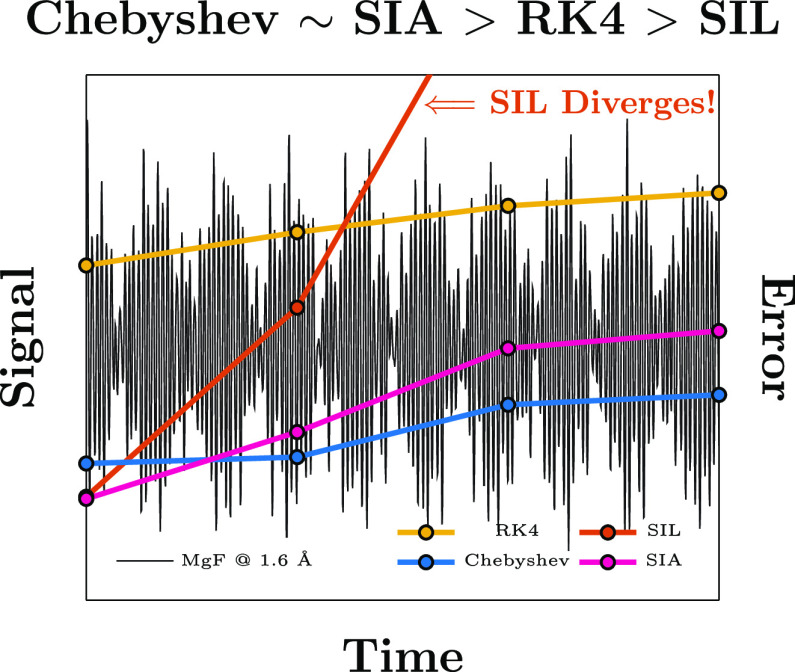

With a growing demand for time-domain simulations of correlated many-body systems, the development of efficient and stable integration schemes for the time-dependent Schrödinger equation is of keen interest in modern electronic structure theory. In this work, we present two approaches for the formation of the quantum propagator for time-dependent equation-of-motion coupled cluster theory based on the Chebyshev and Arnoldi expansions of the complex, nonhermitian matrix exponential, respectively. The proposed algorithms are compared with the short-iterative Lanczos method of Cooper et al. [*J. Phys. Chem. A* 2021 *125*, 5438–5447], the fourth-order Runge–Kutta method, and exact dynamics for a set of small but challenging test problems. For each of the cases studied, both of the proposed integration schemes demonstrate superior accuracy and efficiency relative to the reference simulations.

## 1 Introduction

In recent years, there has been renewed interest in the development of efficient numerical methods to study the quantum dynamics of correlated electrons in molecular and materials systems (see, e.g., refs. (1),^2^ and references therein). Under particular approximations, it is possible to circumvent the direct solution of the time-dependent Schrödinger equation (TDSE) in favor of time-dependent perturbation theory (or “frequency-domain” methods), which aims to implicitly access quantum dynamics through probing the spectral structure of the Hamiltonian operator. In the context of electronic structure theory, these approaches include linear-response,^3−5^ polarization propagator,^6−8^ and equation-of-motion^9−12^ methods, among others.^13−15^ While these methods can often be a powerful tool for the simulation and prediction of observable phenomena such as spectroscopies, their veracity depends on the applicability of their various approximations to accurately characterize queried physical conditions. Further, the vast majority of these perturbative methods serve to access the *equilibrium* behavior of electronic dynamics, leaving nonequilibrium phenomena, such as charge migration,^16^ inaccessible. From a theoretical perspective, time-domain simulations do not suffer from these deficiencies and may be straightforwardly extended to nonperturbative and nonequilibrium regimes.^1,2^

Given the ability to faithfully represent physical conditions by a chosen Hamiltonian, wave function ansatz, and initial condition, the primary challenges of time-domain electronic structure methods are practical rather than theoretical. In contrast to frequency-domain methods which trade the problem of temporal dynamics for the tools of numerical linear algebra,^17−22^ time-domain methods require explicit integration of the TDSE, which is generally more resource-intensive. For hermitian discretizations of molecular Hamiltonians, such as Hartree–Fock (real-time time-dependent HF, RT-TDHF^23,24^), density functional theory (RT-TDDFT),^25^ and configuration interaction (TD-CI),^26−29^ significant research effort has been afforded to the development of efficient numerical methods to integrate the TDSE.^30,31^ In particular, approximate exponential integrators based on polynomial (Chebyshev^30,32−35^) and Krylov subspace (short-iterative Lanczos,^36^ SIL) expansions of the quantum propagator are among the most widely used integration techniques for hermitian quantum dynamics. Exponential integrators are powerful geometric techniques for the solution of linear ordinary differential equations (ODE), such as the TDSE, as they preserve their exact flow,^37^ thereby allowing for much larger time-steps than simpler, nongeometric integrators such as the fourth-order Runge–Kutta method (RK4). In addition, these methods may also be formulated in such a way as to only require knowledge of the action of a matrix–vector product,^30,38−40^ thereby avoiding explicit materialization of the Hamiltonian matrix, which is generally large for correlated many-body wave functions.

The situation is significantly more complex for nonhermitian Hamiltonian discretizations such as those arising from coupled-cluster (CC) theory (see ref (41). for a recent review). Due to its simplicity and low memory requirement, RK4 has generally been the integrator of choice for time-domain CC methods in the recent past.^41^ Symplectic,^42−44^ multistep,^45^ and adaptive^46^ integrators for time-domain CC methods have been developed and have yielded significant efficiency improvements over their nonsymplectic counterparts. Exponential Runge–Kutta integrators have been explored in the context of nonlinear time-dependent CC theory (TD-CC)^47^ but have yet to see wider adoption. Recently, Cooper et al.^48^ suggested an approximate exponential integration scheme for time-dependent equation-of-motion CC theory (TD-EOM-CC)^29,41,43,49−52^ based on the hermitian SIL method to efficiently generate linear absorption spectra for molecular systems. Despite being valid only for hermitian matrices, the proposed SIL approach was demonstrated to produce sufficiently accurate spectra with relatively low subspace dimensions. However, the ability of this scheme to produce faithful, long-time dynamics within TD-EOM-CC has not been assessed and is unlikely due to its hermitian ill-formation. In this work, we pursue the development, application, and assessment of polynomial and nonhermitian Krylov subspace (short-iterative *Arnoldi*, SIA) methods, previously considered for hermitian Hamiltionians,^30,32−35,38,40^ for the complex matrix exponential to enable the efficient and accurate simulation of TD-EOM-CC.

The remainder of this work is organized as follows. In Section 2.1, we review the salient aspects of TD-EOM-CC theory relevant to the development of efficient exponential integrators. In Sections 2.2 and 2.3, we examine the properties of exact and approximate dynamics for the TD-EOM-CC ODE and present the developed integration schemes based on the Chebyshev (Section 2.3.1) and SIA (Section 2.3.2) expansions of the complex matrix exponential. In Section 3, we apply the developed integration schemes to a set of small test problems and compare their veracity with exact dynamics, as well as previously employed SIL and RK4 methods. We conclude this work in Section 4 and offer an outlook on future directions for approximate exponential integrator development in TD-EOM-CC in the years to come.

## 2 Theory and Methods

### 2.1 TD-EOM-CC Theory

TD-EOM-CC theory is a general time-domain reformulation of many-body quantum mechanics capable of simulating the dynamics of both time-dependent^29,41,49,50^ and time-independent^51,52^ Hamiltonians. In this work, we consider the moment-based formulation^51^ of TD-EOM-CC to compute the spectral function
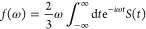
1where *S*(*t*) = ⟨M̃(0)|*M*(*−t*)⟩ = ⟨M̃(*t*)|*M*(0)⟩ is the autocorrelation function. Here, |*M*(*t*)⟩ (⟨M̃(*t*)|) is (the dual of) the time-dependent moment function which describes the propagation of weak perturbations throughout the many-body system. We note for clarity that due to the nonhermiticity of the CC formalism, ⟨M̃(*t*)| is not the complex conjugate of |*M*(*t*)⟩. Additionally, throughout this paper, we chose *S*(*t*) to be ⟨M̃(0)|*M*(−*t*)⟩, although ⟨M̃(*t*)|*M*(0)⟩ is also valid. |*M*(*t*)⟩ (⟨M̃(*t*)|) may generally be described via a linear expansion of (de)excitations from a reference state |0⟩ (typically taken to be HF)
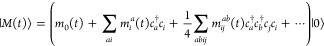
2
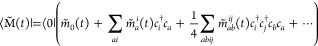
3where *m*_0_ (*m̃*_0_), *m*_*i*_^*a*^ (*m̃*_*a*_^*i*^), and *m*_*ij*_^*ab*^ (*m̃*_*ab*_^*ij*^) are time-dependent (de)excitation amplitudes, *c*_*p*_ (*c*_*p*_^†^) is the Fermionic annihilation (creation) operator associated with the spin–orbital *p*, and the indices *i*, *j*, ... and *a*, *b*, ... denote occupied and virtual spin–orbitals relative to |0⟩. In this work, we truncate eq 2 to include only up to double excitations from the reference, resulting in the TD-EOM-CCSD approach.

Within the TD-EOM-CC formalism, the moment excitation and de-excitation amplitudes obey the following set of coupled, linear-time-invariant (LTI) ODEs^51^
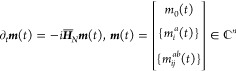
4and their left-hand counterparts
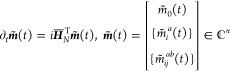
5where  is the nonhermitian, normal-ordered, similarity-transformed Hamiltonian represented in the basis of Slater determinants.^10,11^ From the moment state-vectors, ***m***(*t*) and ***m̃***(t), *S*(*t*) of eq 1 may be evaluated as
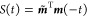
6where we have taken ***m̃***≡***m̃***(0). It is worth mentioning that the TD-EOM-CCSD formalism used here requires propagating only the right- or left-hand moment amplitudes (in this case, the right-hand amplitudes, following eq 4). While eq 1 is perturbatively derived from Fermi’s Golden Rule,^51^ time evolution of |*M*(*t*)⟩ via eq 4 also serves as a useful model for the development of both LTI and non-LTI integration techniques for TD-EOM-CC methods as it formally consists of the same algorithmic components that are required for the simulation of time-dependent Hamiltonians.^29,41,49,50^

When specified as an initial value problem, eq 4 admits an analytic solution
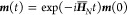
7where exp(−*i****H®***_*N*_*t*) is the quantum propagator and exp is the matrix exponential defined in the canonical way.^53^ We refer the reader to refs (51) and (52), for discussions pertaining to the choices of initial conditions for eq 7 to simulate various spectroscopic properties. In this work, we consider the dipole initial conditions^51^ induced by
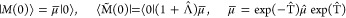
8where T̂ and Λ̂ are the ground-state CC excitation and de-excitation operators (again truncated at double excitation/de-excitations in this work), and μ̂ is a particular component of the electronic dipole operator.

### 2.2 Exact Matrix Exponential

When ***H®***_*N*_ is small enough to be formed explicitly in memory, eq 7 may be directly evaluated as
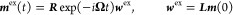
9where  is the diagonal matrix of EOM-CC eigenvalues, , and  are the full, biorthogonal set of corresponding left and right eigenvectors satisfying the equations^10,11^
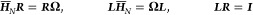
10where  is the identity matrix. As **Ω** is a diagonal matrix, exp(−*i*Ω*t*) is simply the diagonal matrix with entries . Insertion of eq 9 into eq 6 yields the following simple expression for the exact autocorrelation function
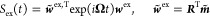
11

As a nonhermitian matrix, ***H***_*N*_ is not guaranteed to have real eigenvalues if the many-electron basis is truncated, and as such, eq 9 (and by extension eq 7) is not guaranteed to be unitary (norm-preserving) and will generally yield dissipative or divergent dynamics along EOM-CC modes with  (see, e.g., a recent study in ref (54).). However, it has been shown that^55,56^, barring suboptimal ground-state CC solutions or the presence of conical intersections, ***H***_*N*_ typically admits a real spectrum representing physical excited states and, thus, eq 9 is unitary in exact arithmetic. Nevertheless, the exact conditions that would enable the prediction of complex eigenvalues a priori are not known; thus, it is paramount for propagation schemes for TD-EOM-CC to properly handle both real and complex spectra. As such, we consider both states of affairs in this work.

### 2.3 Approximate Exponential Integrators

While eq 9 is an exact solution to the LTI TD-EOM-CC dynamics considered in this work, it requires the full diagonalization of ***H®***_*N*_. As the memory requirement associated with the EOM-CCSD ***H***_*N*_ grows *O*(*N*^8^) with system size, full diagonalization is impractical for all but the smallest problems. For some systems, it is possible to integrate the TD-EOM-CC equations in a subspace spanned by a small number of states such that full diagonalization is not required.^29,41,49,50^ However, if a large number of states are required or spectral regions of interest are densely populated or spectrally interior, this approach also becomes impractical.

Matrix exponentiation is a challenging numerical linear algebra problem, and the past half century has yielded a wealth of research into the development of efficient implicit^30,38−40^ and direct^53^ methods both for hermitian and nonhermitian matrices. In this work, we will consider subspace approaches for evaluation of the complex, nonhermitian matrix exponential generally taking the form
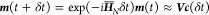
12where  is a *k*-dimensional subspace (with *k* ≪ *n*) generated by the action of −*i****H***_*N*_ onto the current state vector, ***m***(*t*), and  is a time-varying coefficient vector. Given the ability to implicitly form **σ** ← ***H***_*N*_***v*** (i.e., a “σ build”), which is a standard algorithmic component of any EOM-CC implementation,^10,11^ the implementations of eq 12 considered in this work will not require materialization of ***H®***_*N*_ in memory. Within the subspace ansatz, eq 6 becomes
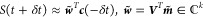
13where ***w̃*** is time-independent for fixed ***V***.

For a particular expansion order *k* and state vector ***m***(*t*), eq 12 will generally be valid for |δ*t*| ≤ |Δ*t*|, where Δ*t* will be referred to as a *macro time-step* in the following. Within this prescription, the total simulation length, , will be partitioned into subintervals  where *t*_0_ = 0, *t*_*i*_ = *t*_*i*–1_ + Δ*t*_*i*_ and Δ*t*_*i*_ is the macro-time-step for the *i*-th interval. The relationship between *k* and Δ*t* is method-dependent and will be discussed for both the Chebyshev and Arnoldi integrators below. Due to the factorization of the time-dependence into ***c***(*t*), a general property of truncated expansions such as eq 12 is in their ability to interpolate within each  without requiring additional σ builds.^30^ This property is particularly advantageous for methods such as EOM-CCSD in which the computational complexity of σ formation scales *O*(*N*^6^) with system size.^10,11^ For each , a single ***V*** is computed and the propagator may be interpolated to arbitrary temporal resolution by varying the corresponding coefficients. For each of the intermediate time intervals (*i* > 0), the approximation of ***m***(*t*_*i*+1_) generated from the end point of  is used as the starting vector to generate ***V*** for .

#### 2.3.1 Chebyshev Time Integration

The use of the Chebyshev expansion to evaluate the quantum propagator for hermitian Hamiltonians is well established and is among the most efficient known strategies for integrating LTI variants of the TDSE.^30,32−35^ In this work, we demonstrate that this approach is also applicable to nonhermitian Hamiltonians with real or complex spectra. In the present treatment, we work with modified Chebyshev polynomials of the first kind, {Φ_*p*_}, given by the recurrence
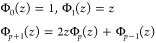
14In the Chebyshev basis, the TD-EOM-CC propagator acting on a general vector ***v*** may be exactly expanded as^30,32^
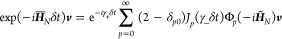
15where , ω_min/max_ are the minimum/maximum eigenvalues of ***H®***_*N*_, δ_*k*0_ is a Kronecker delta, *J*_*p*_ is the *p*-th Bessel function of the first kind, and ***H̃***_*N*_ = γ_–_^–1^(***H®***_*N*_–γ_+_***I***) is an auxiliary matrix that scales the spectrum of ***H®***_*N*_ from [ω_min_, ω_max_] → [−1, 1] such that the image of Φ_*p*_ remains within the unit disk. Practically, ***H̃***_*N*_ need not be formed explicitly (see Alg. 1) and γ_±_ need not be computed from exact eigenvalues and can be approximated using standard techniques.^57−61^

**Figure 1:**
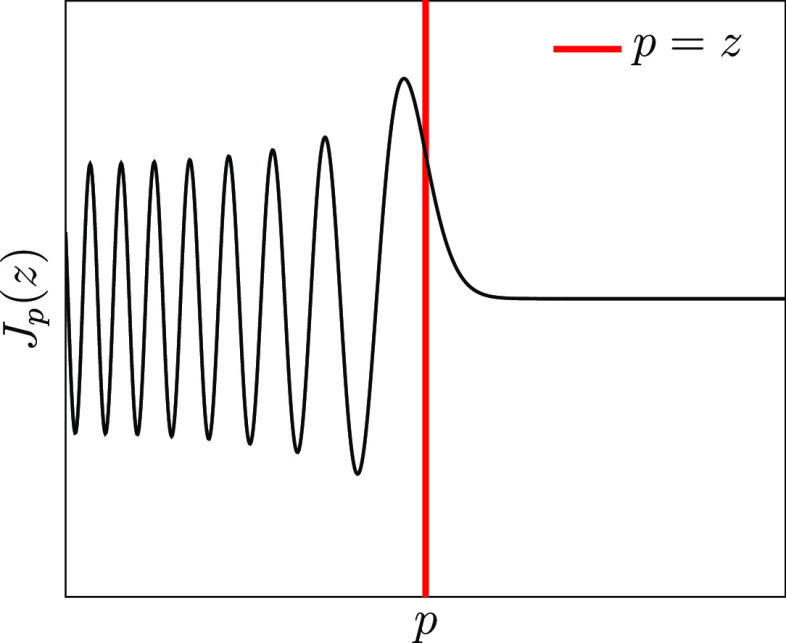
Graphical depiction of the order decay behavior of Bessel functions of the first kind for fixed argument. The function is highly oscillatory for *p* < *z* but decays exponentially for *p* > *z*.

In practice, the sum in eq 15 is truncated to a finite order *k*, yielding a compact representation of the propagator in the Chebyshev basis, ***V***_cheb_ = [***v***_0_^cheb^,***v***_1_^cheb^,***v***_2_^cheb^,...,***v***_*k*–1_^cheb^], given by
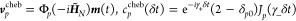
16

For real-valued spectra, the truncation error at the interval end point (*t* + Δ*t*) of the Chebyshev expansion can be shown^62,63^ to be bounded by
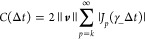
17For fixed argument, *J*_*p*_(*z*) is highly oscillatory for *p* < *z* but decays exponentially for *p* > *z*, as depicted in Figure 1. We note that for even (odd) *p*, *J*_*p*_ is an even (odd) function of about zero. Therefore, for *p* sufficiently larger than |γ_–_Δ*t*|, we may approximate *C*(Δ*t*) ≈ 2∥***v***∥|*J*_*p*_(γ_–_Δ*t*)|. Given a desired step size, Δ*t*_cheb_, and an error threshold ε^cheb^, we may use this approximation to select *k* > |γ_–_Δ*t*_cheb_| such that . We note that due to the fact that the many-body Hamiltonian is an unbounded operator, the spectral radius of ***H®***_*N*_ is known to grow superlinearly with basis and system size. As such, it should be expected that for large systems, the number of Chebyshev terms required to achieve an accurate approximation of the propagator will grow at a commensurate rate.

For complex spectra, the convergence analysis for the Chebyshev expansion becomes slightly more challenging as the uniform convergence of |*J*_*p*_(α)Φ_*p*_(*z*)| with respect to increasing *p* is only a property held by *z* ∈ [−*i*, *i*].^64^ Outside of this domain, |Φ_*p*_| grows exponentially. However, as the complex exponential is holomorphic, eq 15 is guaranteed to converge absolutely in all of . As the typical manifestation of complex eigenvalues in EOM-CC spectra yields imaginary parts much smaller in magnitude than the spectral radius of ***H®***_*N*_ (see, e.g., refs (54)–^56^) and given that the spectrum is already scaled by this radius (further reducing this magnitude), it is expected that these small imaginary components lie close enough to [−*i*, *i*] that eq 17 remains a valid metric by which one may derive the required order of the Chebyshev expansion. We examine this behavior for complex spectra in Section 3.
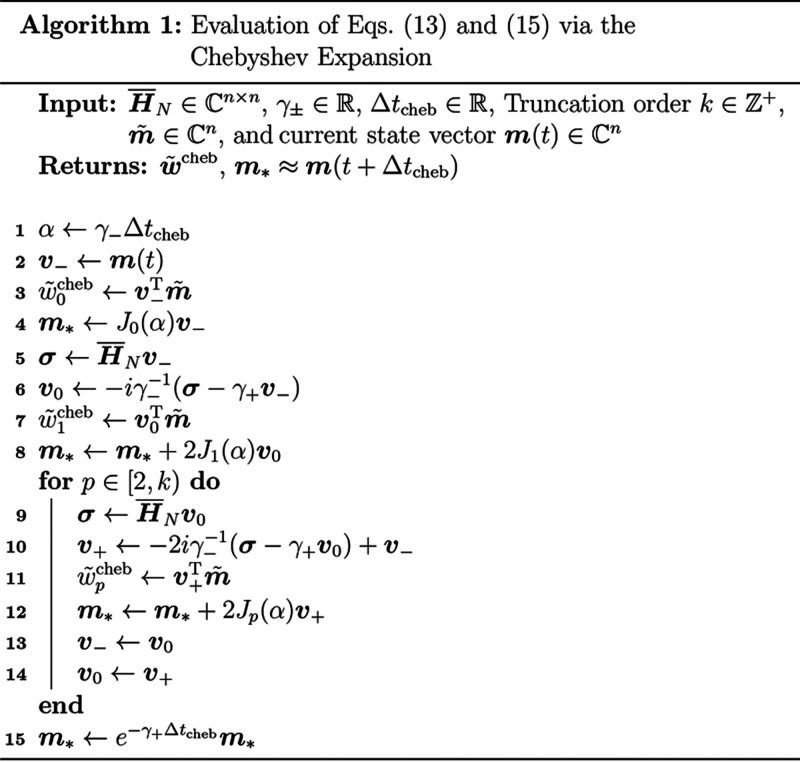


As Δ*t*_cheb_ is fixed,  may be evenly partitioned into  intervals. The Chebyshev subspace vectors may be efficiently evaluated using only *k* σ-builds (Alg. 1); thus, the total σ-build cost for this method is . Another important aspect of the Chebyshev method is that because the expressions in eq 16 are analytic, one need not materialize ***V***_cheb_ in memory. Instead, one may evaluate ***w*~**^cheb^ = ***V***_cheb_^T^***m̃*** (eq 13) directly as the subspace is generated, as is shown in Alg. 1, thus changing the memory requirement from *O*(*kn*) to *O*(3*n*). As it is often the case that one requires high-order Chebyshev polynomials (≫ 3) to accurately approximate the matrix exponential, this realization leads to a drastic reduction in memory consumption for large systems.

#### 2.3.2 Short Iterative Arnoldi Time Integration

Considering the spectral decomposition of the exact propagator given in Section 2.2, it is expected that the Chebyshev method discussed in Section 2.3.1 will be most effective when Ω is nearly uniformly distributed within [ω_min_, ω_max_] because the Chebyshev basis minimizes the uniform error norm. If Ω is clustered, Krylov subspace techniques for the formation of the exponential propagator are often more effective.^38^ The basic principle behind Krylov approximation techniques for matrix functions is rooted in the generation of a *k*-dimensional, orthonormal basis, ***V***_krlv_ = [***v***_0_^krlv^,***v***_1_^krlv^,...,***v***_*k*–1_^krlv^], for the Krylov subspace
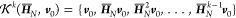
18where  is an arbitrary vector with ∥***v***_0_∥ = 1. Given **V**_krlv_, one may form a subspace-projected Hamiltonian
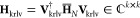
19and approximate the action of the matrix exponential as^38^
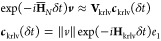
20where *e*_1_ is the first column of a *k* × *k* identity matrix and **V**_krlv_ is the Krylov subspace generated from ***v***_0_ = ***v***/∥***v***∥. Given that *k* ≪ *n*, the exponential in eq 20 may be efficiently evaluated via eq 9.

For hermitian matrices, **V**_krlv_ can be efficiently generated by the Lanczos iteration,^65^**H**_krlv_ is a tridiagonal matrix, and both **H**_krlv_ and **V**_krlv_ may be formed implicitly via a simple three-term recursion. For the approximation of the propagator, this approach has come to be known as the short-iterative Lanczos (SIL) method.^36^ Here, we present an analogous scheme for the exponential propagator based on the Arnoldi iteration,^65,66^ which is a general Krylov subspace technique which extends to both hermitian and nonhermitian matrices with both real- and complex-valued spectra. We will refer to this approach as the short-iterative Arnoldi (SIA) method in the following. Instead of a tridiagonal matrix, the Arnoldi method produces an upper Hessenberg matrix via the recursion
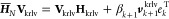
21where *e*_*k*_ is the *k*-th column of the *k* × *k* identity matrix and β_*k*+1_***v***_*k*+1_^krlv^ is the residual
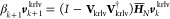
22with ∥***v***_*k*+1_^krlv^∥ = 1. If ***H***_*N*_ were a hermitian matrix, **H**_krlv_ would be tridiagonal and **V**_krlv_ would span the same subspace as the one produced by the Lanczos iteration in exact arithmetic.
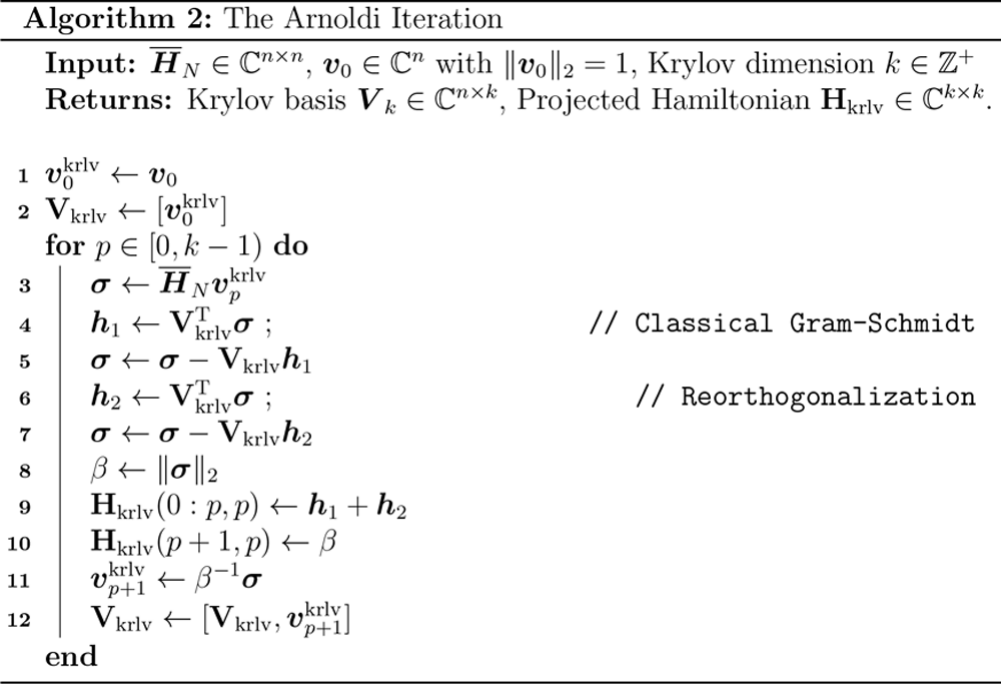


Much like the Lanczos iteration, **H**_krlv_ may also be formed incrementally via the Arnoldi iteration, as shown in Alg. 2. However, unlike the 3-term recurrence used in the Lanczos method, the Arnoldi iteration requires *explicit* orthogonalization of newly produced subspace vectors as opposed to the implicit orthgonalization generated by Lanczos. As the Arnoldi method is guaranteed to produce an orthonormal basis via explicit orthogonalization, it is often more numerically stable even for hermitian problems.^67−69^ In this work, we have utilized the classical Gram-Schmidt method with reorthogonalization to perform the explicit basis orthogonalization.^70^ There exist nonhermitian extensions of the Lanczos method^71^ which produce simultaneous, biorthogonal approximations for the left- and right-hand eigenspaces of nonhermitian matrices and have seen successful applications in both frequency-domain CC applications^19^ as well as state selection for TD-EOM-CC.^50^ However, the biorthogonalization requirements of these methods can often be numerically unstable,^72−74^ and as such, we expect the Arnoldi method to yield superior numerical stability in finite precision.^75^

It has been shown^38^ that the error produced by eq 20 can be bounded by the right-hand side of the following inequality
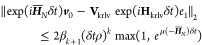
23where *μ*(***H®***_N_) is the largest eigenvalue of (***H®***_*N*_ + ***H®***_*N*_^†^)/2 and . Although tighter bounds can be found,^40^ the bound given in (23) is more instructive. It shows that the approximation error made in an Arnoldi time integrator depends on the departure of **V**_krlv_ from an invariant subspace of ***H®***_*N*_, which is measured by β_*k*+1_, the step size or time window δ*t* as well as the spectral radius of ***H***_*N*_, measured by ρ and μ(***H***_*N*_).

Unlike the Chebyshev method, where the expansion coefficients are known ahead of time, the coefficients for SIA are related to the spectrum of **H**_krlv_, which itself is dependent on ***v*** [the current state vector, ***m***(*t*), in the context of eq 12]. As such, it is canonical to adopt a dynamic time-stepping approach where the Krylov subspace dimension (*k*) is fixed before the simulation and each Δ*t*_*i*_ corresponding to  is determined dynamically throughout the time propagation. As eq 23 is only a loose bound, its practical ability to determine Δ*t* is limited. Given that the Arnoldi method produces successively more accurate Krylov subspaces with increasing *k*, a more practical error bound is given by *c*_*k*_^krlv^(Δ*t*), which measures the potential for projections of the exact matrix exponential onto vectors outside the Krylov subspace. Therefore, as has been successfully applied to the SIL method,^48^ a reasonable choice for the step size is the largest Δ*t* such that |*c*_*k*_^krlv^(Δ*t*)|<ε^krylov^, where  is a chosen error threshold.

Another side effect of the nonanalytic nature of the SIA coefficients is that, unlike **V**_cheb_, **V**_krlv_ must be materialized in memory and eqs 13 and 15 must be evaluated explicitly. As such, the memory requirement associated with SIA will grow *O*(*kn*) with the basis dimension. However, as will be demonstrated in Section 3, the SIA method will generally require fewer σ builds than the Chebyshev method to achieve commensurate integration accuracy.

## 3 Results

**Figure 2:**
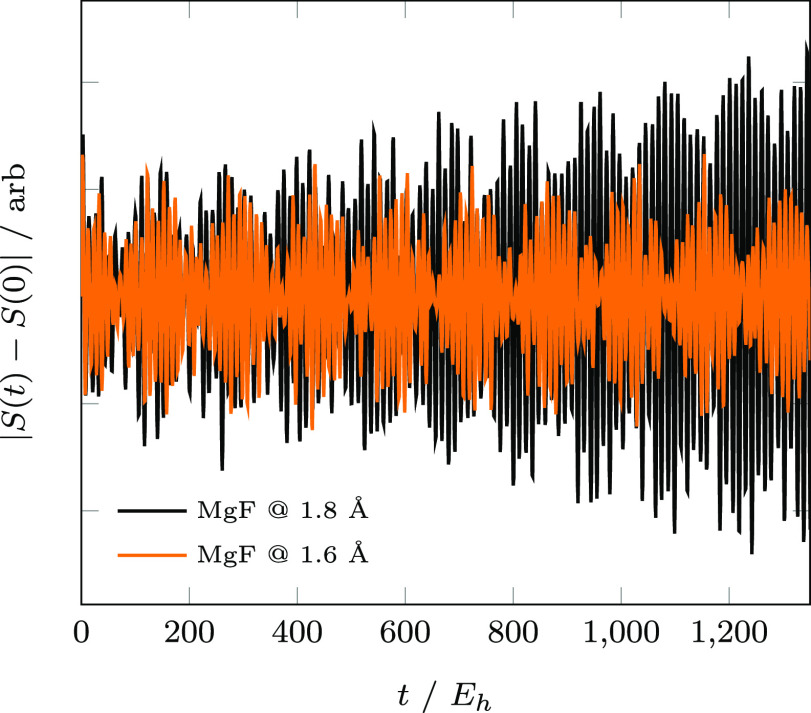
Absolute value of the autocorrelation function for MgF at 1.6 and 1.8 Å internuclear separation. The EOM-CC spectrum of the former only exhibits real eigenvalues, while that of the latter exhibits a complex eigenvalue pair with imaginary component ≈6*mE*_h_.

To assess the efficacy of the Chebyshev and SIA TD-EOM-CC integrators developed in this work, we compare the accuracy and efficiency of these methods for three test systems, N_2_ (1.1 Å) and MgF (1.6 and 1.8 Å), relative to exact dynamics (eq 9) as well as RK4 and the TD-EOM-CC SIL method of ref (48). Each of these systems were treated at the EOM-CCSD level of theory with the minimum STO-3G basis set^76,77^ to allow for practical comparisons with exact dynamics. All ground-state CC calculations were performed using a prototype Python implementation interfaced with the HF and integral transformation routines in the Psi4 software package^78^ and geometries were aligned along the *z*-Cartesian axis without the use of point-group symmetry. At their respective geometries, N_2_ and MgF @ 1.6 Å exhibit real-valued EOM-CC spectra while MgF @ 1.8 Å exhibits a pair of complex conjugate eigenvalues with their imaginary components being ≈0.6 mE_h_. All simulations in this work were performed using ε^cheb^ = 10^–16^ and ε^krylov^ = 10^–6^ (for both SIL and SIA) for a duration of  (≈32 fs). Exact results for the MgF simulations are given in Figure 2 to exemplify the temporal behavior *S*(*t*) in the presence of complex eigenvalues. MgF @ 1.6 Å exhibits a constant signal profile, while MgF @ 1.8 Å exhibits exponential growth. We refer the reader to ref (54). for a more comprehensive discussion of this behavior.

**Figure 3:**
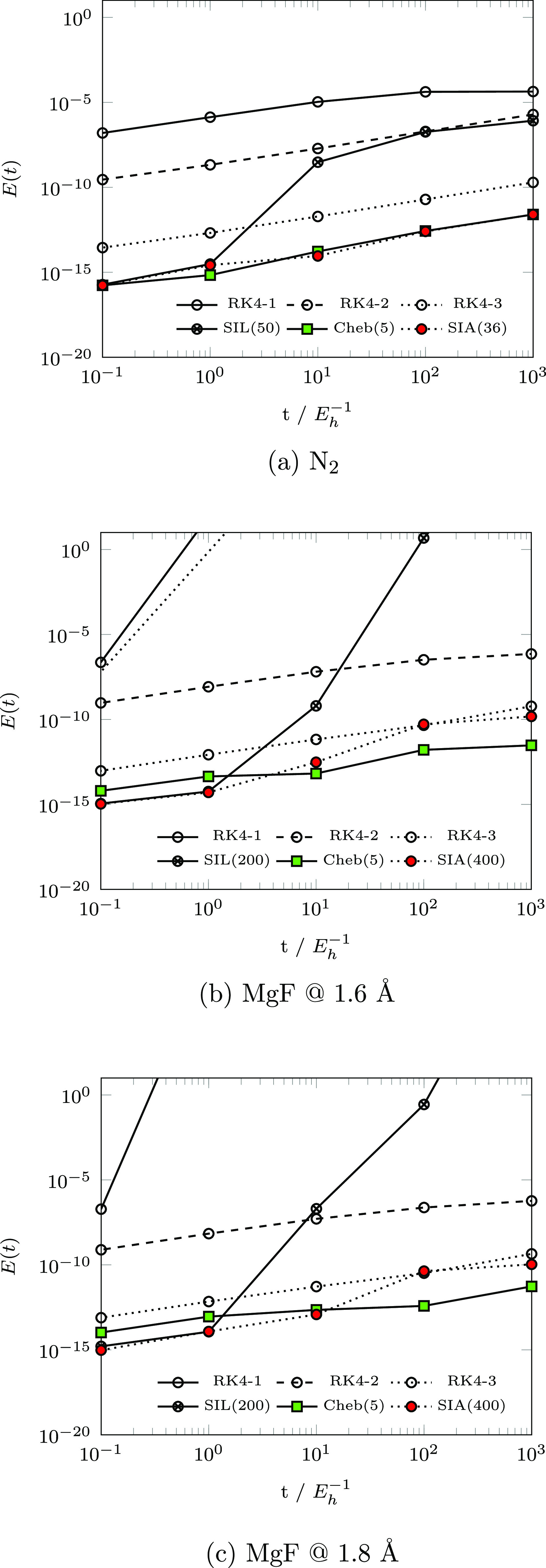
Accumulated *S*(*t*) errors for RK4, Chebyshev, SIA, and SIL.

First, we examine the temporal error accumulation in the autocorrelation function (eq 1) using the normalized root-mean-square-deviation (rmsd) metric
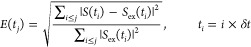
24where *S*_ex_ is given in eq 11 and δ*t* is the temporal resolution of the integrated time series. For the Chebyshev, SIA, and SIL integrators, δ*t* = 0.05*E*_h_^–1^. As the temporal resolution and step size coincide for RK4, we have compared our methods with 3 different RK4 step sizes to illustrate convergence: RK4–1 (δ*t* = 0.05*E*_h_^–1^), RK4–2 (δ*t* = 0.01*E*_h_^–1^), and RK4–3 (δ*t* = 0.001*E*_h_^–1^). In the following, we will use  (i.e., the total accumulated autocorrelation error) as a global error metric to assess each integrator’s relative accuracy. Figure 3 illustrates the accumulated autocorrelation error for each of the integrators considered. Parameters for Chebyshev (Δ*t*_cheb_), SIA (*k*), and SIL (*k*) simulations in Figure 3 were selected to minimize  for each method. For N_2_, the Chebyshev, SIA, and RK4 integrators exhibit near constant error accumulation over the full simulation. SIL exhibits a sharp error increase between 1 and 10 *E*_h_^–1^ which is of the same order as ε^krylov^. For *k* = 36, SIA yields an invariant subspace up to an error of *O*(ε^krylov^), and as such, the entire simulation  can be performed using a single Krylov subspace. At both geometries, SIL and RK4-1 diverge for MgF, while Chebyshev, SIA, RK4-2, and RK4-3 exhibit error accumulation characteristics similar to those observed for N_2_. However, unlike in the N_2_ case, SIA does not yield an invariant subspace even with the largest subspace of *k* = 400, and thus multiple Krylov subspaces must be generated over the course of the simulation. As such, error *O*(ε^krylov^) is compounded at each macro-time-step, which explains the overtaking of SIA by Chebyshev in the long-*t* limit. We also note that the error accumulation profiles for both MgF @ 1.6 Å and MgF @ 1.8 Å are nearly identical with the exception that SIL diverges marginally faster for the system with complex eigenvalues (1.8 Å) than that with real eigenvalues (1.6 Å). This numerical experiment confirms the efficacy of the Chebyshev and SIA schemes in the presence of complex eigenvalues in the EOM-CC spectrum, and thus, we will focus on the systems with real eigenvalues (i.e., N_2_ and MgF @ 1.6 Å) for the remainder of the numerical experiments.

**Figure 4:**
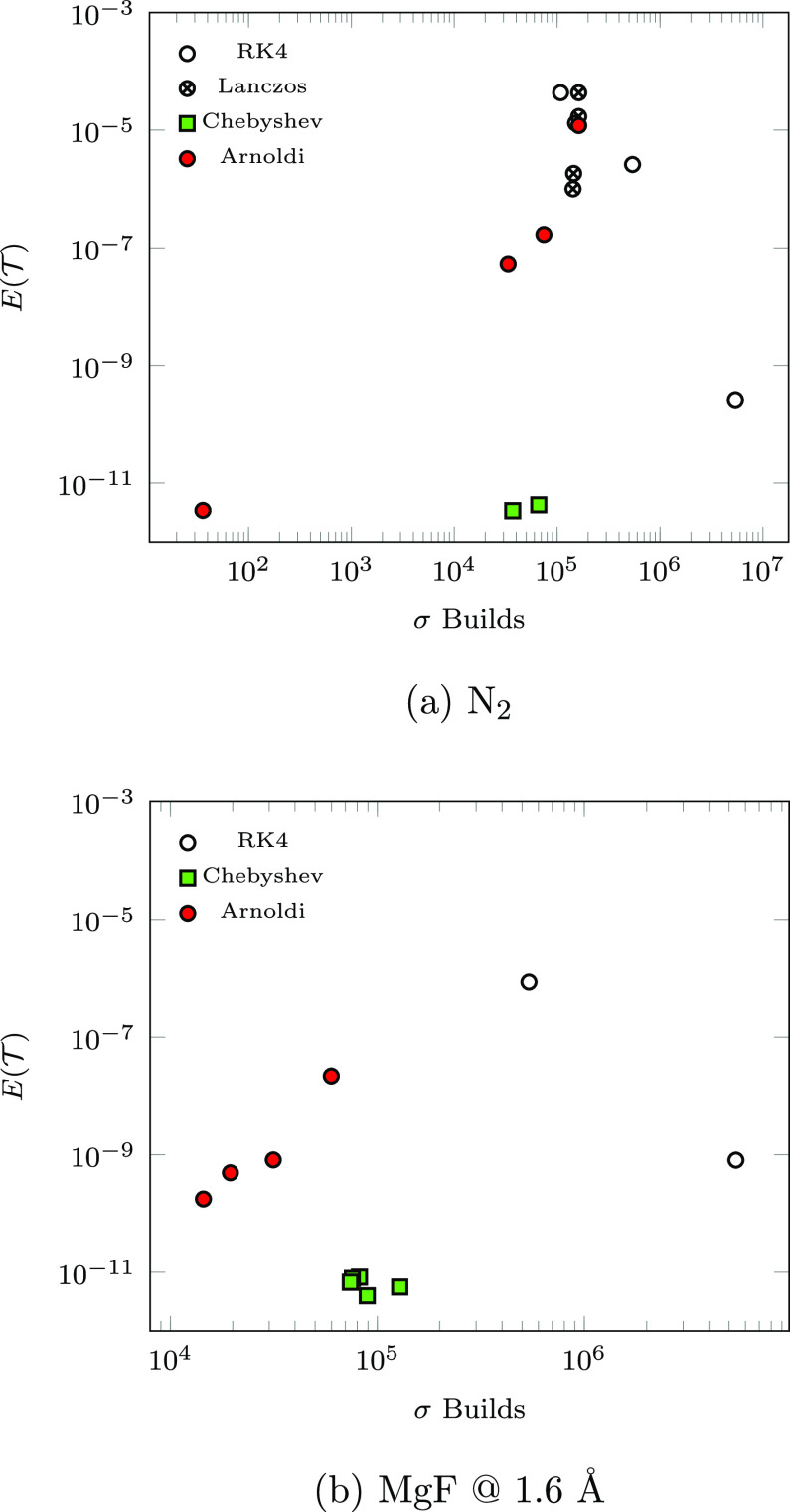
Cost-to-accuracy comparison for RK4, Chebyshev, SIA, and SIL.

**Figure 5:**
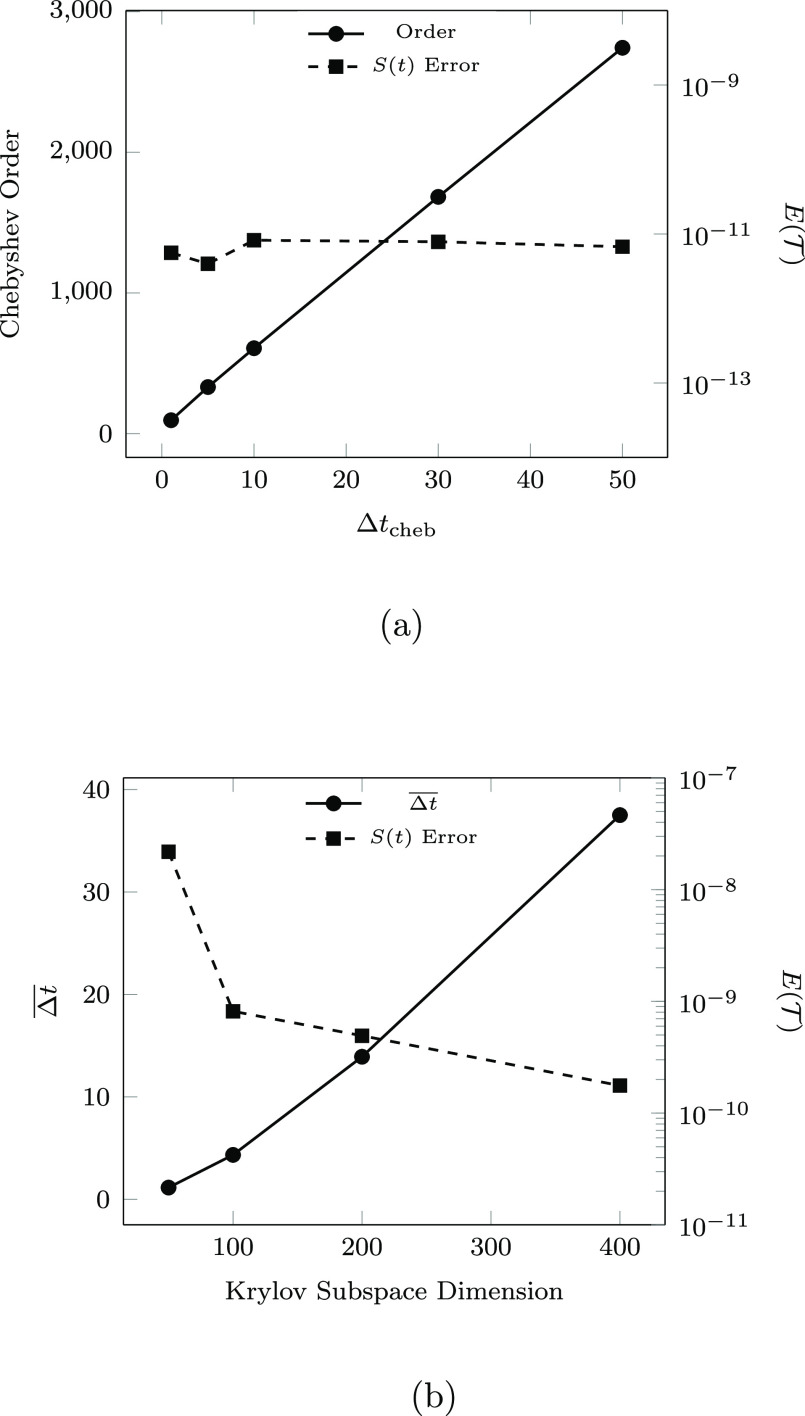
Assessment of the variance of cost and accuracy of (a) Chebyshev and (b) SIA integrators as a function of parameter selection for MgF at 1.6 Å. SIA results are presented as the average time-step  as a function of *k*.

Figure 4 presents the cost-to-accuracy ratio, characterized by  as a function of σ builds emitted by each integrator, for a range of parameter choices. For N_2_ (MgF @ 1.6 Å), Chebyshev results were obtained for Δ*t*_cheb_ ∈ {1, 5} (Δ*t*_cheb_ ∈ {1, 5, 10, 30, 50}). As discussed in Section 2.3.1, the number of required σ builds for the Chebyshev is fixed at  and *m*_cheb_ generally increases as a function of Δ*t*_cheb_. This behavior is shown explicitly for MgF @ 1.6 Å in Figure 5. For both systems studied, neither  nor the total number of σ-formations is significantly affected by increasing Δ*t*_cheb_.

SIA results were obtained for N_2_ (MgF @ 1.6 Å) with *k* ∈ {5, 10, 20, and 36} (*k* ∈ {50, 100, 200, 400}). As is shown, the achievable time-step (σ build count) subject to ε^krylov^ is (inversely) proportional to *k* and, thus, the SIA and SIL data points in Figure 4 are plotted in order of decreasing *k*. Unlike the Chebyshev method, the accuracy of SIA consistently improves with increased *k*, and thus, *k* should be maximized subject to available memory resources to improve both accuracy and efficiency of the SIA method.

For N_2_, SIL results were also obtained with *k* ∈ {5, 10, 20, 36, 50} for a direct order-by-order comparison with SIA. At each order, SIA achieves better accuracy over SIL by between 2 and 3 orders of magnitude and requires >50% fewer σ builds in cases where SIA is able to take time-steps larger than δ*t* (*k* ≥ 10). This is due to the fact that the Arnoldi method generates a faithful Krylov subspace representation ***H̅***_*N*_, while the Lanczos method, being only valid for hermitian matrices, does not. This fact is particularly apparent in SIA’s generation of an invariant subspace for *k* = 36, while SIL fails to demonstrate similar convergence.

For all problems considered, the proposed SIA and Chebyshev integrators exhibit superior accuracy and efficiency over analogous SIL and RK4 simulations. While it is possible for RK4 to yield reasonable accuracy at small time-steps (RK4-3), these simulations require an excessive number of σ builds and would not be practical for the simulation of realistic TD-EOM-CC problems.

## 4 Conclusions

In this work, we have presented two approximate exponential time-integrators for the TD-EOM-CC theory based on Chebyshev and Arnoldi (SIA) expansions of the quantum propagator. The efficacies of these integrators were demonstrated via comparison with the exact exponential dynamics for three small test problems with both real and complex EOM-CC spectra. The Chebyshev and SIA integrators were demonstrated to yield superior accuracy and efficiency when compared to RK4 and the recently developed SIL method for TD-EOM-CC.^48^ As both of the presented methods are built from the standard algorithmic components required for any implementation of (TD-)EOM-CC, the implementation of these methods has a low barrier for entry and holds the potential to yield significant performance and accurate improvements for these simulations in the future.

The practical application of the presented schemes requires consideration of the balance between the desired integration accuracy and available computational resources. If memory capacity allows, the SIA method would be preferred for most chemistry applications due to its systematic improvability with respect to truncation order. However, the memory requirement of SIA quickly becomes prohibitive for large problems, and the explicit orthogonalization requirement complicated efficiently distributed memory implementations. In these instances, the Chebyshev method would be preferred due to its low memory requirement and the simplicity of its implementation. However, given the noted dependence of the required Chebyshev order on the spectral radius of ***H̅***_*N*_, the SIA method may still be preferable for particularly large systems due to its ability to simultaneously and compactly approximate the extreme ends of the spectrum.

While the results presented in this work have focused on the moment-based formalism of TD-EOM-CC, the presented efficacy experiments serve as an important proof of concept to demonstrate the proposed methods for general TD-EOM-CC simulations. Future work to extend these methods to large-scale TD-EOM-CC simulations is currently being pursued by the authors. Further, extension of these methods for use with time-dependent Hamiltonians, such as those required to study field-driven dynamics of molecular systems, is currently under development. While the moment-based formalism considered in this work requires only the propagation of the right-hand (or left-hand) EOM-CC state, modeling field-driven dynamics with a time-dependent Hamiltonian will require propagating both the right- and left-hand states. As such, the performance of the integrators we have presented should be re-evaluated for this use case.
